# Beyond Microsatellite Instability: Evolving Strategies Integrating Immunotherapy for Microsatellite Stable Colorectal Cancer

**DOI:** 10.1007/s11864-021-00870-z

**Published:** 2021-06-10

**Authors:** Federica Pecci, Luca Cantini, Alessandro Bittoni, Edoardo Lenci, Alessio Lupi, Sonia Crocetti, Enrica Giglio, Riccardo Giampieri, Rossana Berardi

**Affiliations:** Clinical Oncology, Università Politecnica delle Marche, AOU Ospedali Riuniti, Via Conca 71, 60126 Ancona, Italy

**Keywords:** Immune checkpoint inhibitors, Colorectal cancer, Proficient DNA mismatch repair, Tumor microenvironment

## Abstract

Advanced colorectal cancer (CRC) is a heterogeneous disease, characterized by several subtypes with distinctive genetic and epigenetic patterns. During the last years, immune checkpoint inhibitors (ICIs) have revamped the standard of care of several tumors such as non-small cell lung cancer and melanoma, highlighting the role of immune cells in tumor microenvironment (TME) and their impact on cancer progression and treatment efficacy. An “immunoscore,” based on the percentage of two lymphocyte populations both at tumor core and invasive margin, has been shown to improve prediction of treatment outcome when added to UICC-TNM classification. To date, pembrolizumab, an anti-programmed death protein 1 (PD1) inhibitor, has gained approval as first-line therapy for mismatch-repair-deficient (dMMR) and microsatellite instability-high (MSI-H) advanced CRC. On the other hand, no reports of efficacy have been presented in mismatch-repair-proficient (pMMR) and microsatellite instability-low (MSI-L) or microsatellite stable (MSS) CRC. This group includes roughly 95% of all advanced CRC, and standard chemotherapy, in addition to anti-EGFR or anti-angiogenesis drugs, still represents first treatment choice. Hopefully, deeper understanding of CRC immune landscape and of the impact of specific genetic and epigenetic alterations on tumor immunogenicity might lead to the development of new drug combination strategies to overcome ICIs resistance in pMMR CRC, thus paving the way for immunotherapy even in this subgroup.

## Introduction: the role of immune checkpoint inhibitors in dMMR CRC

MLH1, MSH2, MSH6, PMS2, and EPCAM genes encode for proteins involved in DNA mismatch repair (MMR). Alterations of at least one of these genes make identification and repair of spontaneous mutations impossible, leading to rapid accumulation of other variants, high microsatellite instability (MSI-H), and increased tumor mutational load [1–3]. Therefore, according to their mutational status, colorectal cancer (CRC) can be divided into two groups: mismatch-repair-deficient (dMMR) and MSI-H tumors (MSI-H-dMMR CRC) and mismatch-repair-proficient (pMMR) and microsatellite instability-low (MSI-L)/microsatellite stable (MSS) tumors (MSI-L/MSS-pMMR CRC). In particular, approximately 15% of all CRC has a MSI-H-dMMR signature [4], and only 3–4% of patients with metastatic CRC (mCRC) detains MSI-H-dMMR status, due to germline, somatic, or epigenetic inactivation MMR genes [1]. In 2015, Le et al. showed that pembrolizumab, a programmed cell death protein 1 (PD1) inhibitor, had a different activity in CRC based on MMR status; the immune-related objective response rate (ORR) was 40%, and progression-free survival (PFS) rate was 78% in MSI-H-dMMR CRC and 0% and 11% respectively for MSI-L/MSS-pMMR CRC [5]. Investigators in the KEYNOTE-177 (a phase 3, open-label trial) compared the efficacy of first-line pembrolizumab monotherapy vs standard chemotherapy in 307 patients affected by MSI-H-dMMR mCRC: Pembrolizumab was superior to chemotherapy in terms of PFS (median 16.5 months vs 8.2 months) (*p* = 0.0002) and ORR (43.8% vs 33.1%) [6••]. In 2017, the Checkmate-142 trial assessed the activity of first-line monotherapy with nivolumab, another anti-PD1 inhibitor, in 74 patients affected by MSI-H-dMMR mCRC. At a median follow-up of 12 months, the ORR was 31% and 69% of patients detained disease control for 12 weeks or longer [7]. In the same trial, the combination of nivolumab with ipilimumab, a cytotoxic T lymphocyte antigen 4 (CTLA4) inhibitor, achieved 64% ORR, 9% complete response (CR) rate, and 84% disease control rate (DCR) as first-line treatment for MSI-H-dMMR mCRC patients [8, 9]. Therefore, immune checkpoint inhibitors (ICIs) demonstrated particular efficacy in dMMR mCRC but disappointing results in pMMR [5]. The explanation to that could be found in many mechanisms of resistance to immunotherapy such as the presence of immunosuppressive factors in local tumor microenvironment (TME), downregulation of major histocompatibility complex (MHC) protein expression on tumor cell surface, tumor clonal heterogeneity, and tumor dedifferentiation and stemness [10]. Combination strategies aimed to restore tumor immunogenicity are being developed to overcome immunotherapy resistance [11]. To this regard, similarly to other cancer types [12–14], prognostic and predictive markers beyond MSI profile are needed, to better identify mCRC patents that could benefit from immunotherapy alone or from drug combination [15–17]. The aim of this review is to describe the biological mechanisms of resistance to immune checkpoint inhibitors (ICIs) in MSI-H-pMMR CRC and the therapeutic combination strategies to overcome them.

## Biology beyond immune checkpoint inhibitors resistance in pMMR CRC

**Fig. 1:**
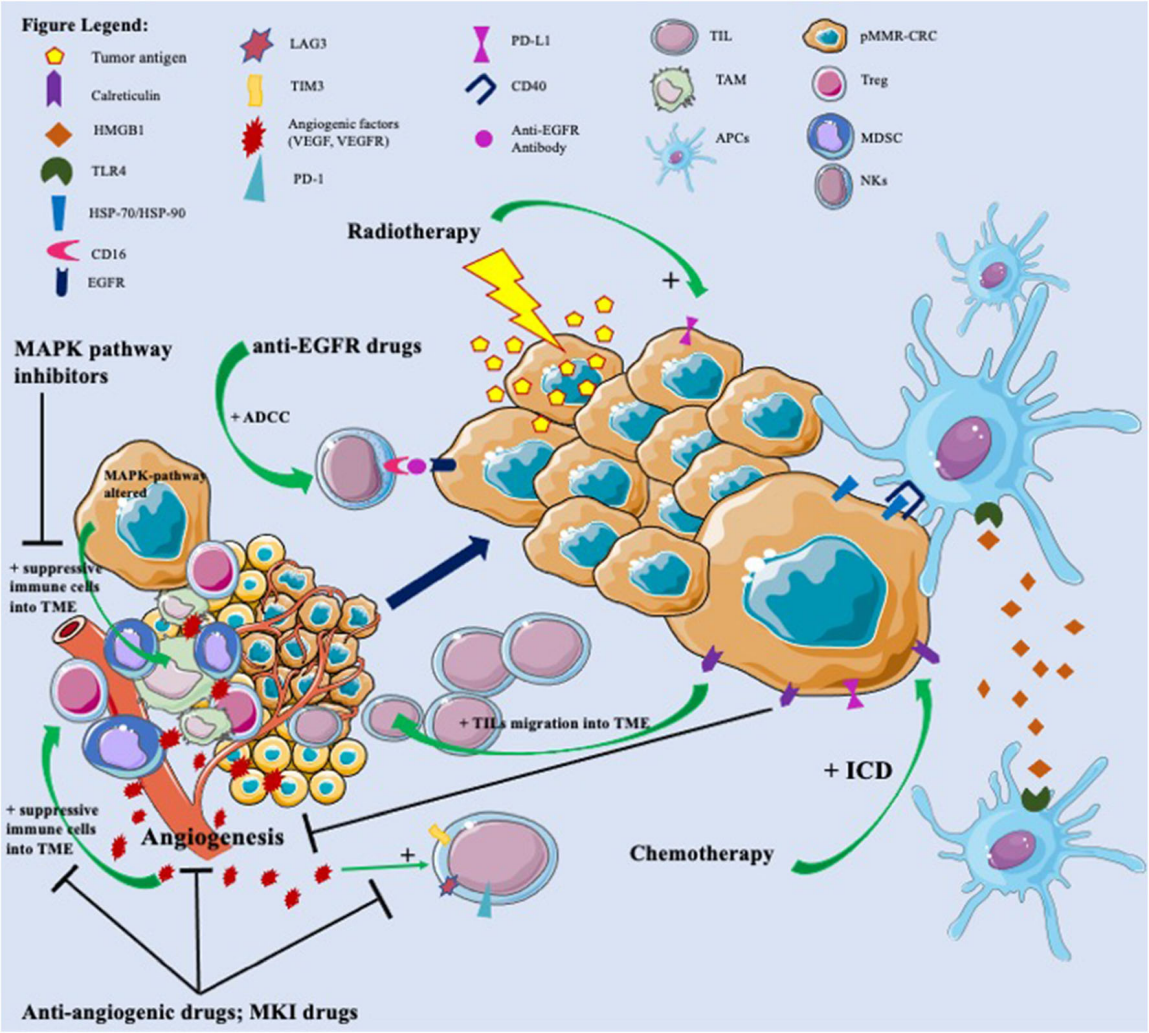
pMMR CRC are characterized by an immune-excluded and immune-suppressive tumor microenvironment (TME), leading to resistance to immune checkpoint inhibitors (ICIs). In fact, tumor-infiltrating lymphocytes (TILs) and antigen-presenting cells (APCs) are located outside of tumor core and inner invasive margin, reducing their direct contact with tumor cells. Inside tumor core, myeloid-derived suppressor cells (MDSCs), tumor-associated macrophages (TAMs), and regulatory T cells (Treg) lead to suppression of immune response against cancer cells. Moreover, pMMR CRC detains low levels of neoantigens, impairing their presentation to CD8+ T cells. In this immune-excluded and immune-suppressive TME, chemotherapy, inducing immunogenic cell death (ICD), promotes exposure on tumor cell surface of calreticulin, able to inhibit tumor angiogenesis and increased TILs inside TME, and of heat shock protein (HSP) 70 and 90, able to bind CD40 on dendritic cells (DCs) and activating them. Moreover, ICD promotes the release from tumor cells of HMGB1 that could bind TLR4 on DCs surface and promote their activation. Radiotherapy leads to increased neoantigen presentation and release from tumor cells and increased expression of PD-L1. Anti-angiogenesis drugs, such as bevacizumab, and other multikinase inhibitors (MKI), such as regorafenib, are able to inhibit the immuno-suppressive effect of VEGF/VEGFR on TME, blocking the infiltration of MDSCs, TAMs, and Treg inside TME and the expression of T cell exhaustion markers (PD-1, LAG3, TIM3). Anti-EGFR drugs, inducing antibody-dependent cell-mediated cytotoxicity (ADCC), lead to cancer cells lysis by natural killer (NK) cells. Finally, alterations of MAPK pathway on cancer cells promote migration of suppressive immune cells inside TME and reduce TILs infiltration; therefore, drugs that target this axis might restore antitumor immune cell activity.

Efficacy of ICIs is related to a wide spectrum of factors, including tumor neoantigens level and presentation, immune cell infiltration and phenotype, and regulatory checkpoint receptors. On the one hand, dMMR CRC harbors a high tumor mutational burden (TMB) that leads to high mutation-associated neoantigen load, higher CD8^+^ cytotoxic T and Th1 helper cells infiltration [15], and high levels of human leukocyte antigen (HLA) proteins. Conversely, pMMR CRC is considered “immune-excluded” and “cold” tumor [18]. Based on T cell density in each tumor spatial compartment (tumor core, inner invasive margin, and outer invasive margin), tumors are defined “cold” when low T cell density is observed in every compartment, “excluded” when high T cell infiltration is present only in the outer invasive margin, and is low in other compartments and “hot” when high T cell density is present both in the core and inner invasive margin [19••]. ICIs efficacy implies direct contact between tumor-infiltrating lymphocytes (TILs), antigen-presenting cells (APCs), and tumor cells to trigger a strong and specific antitumor immune response. This happens in dMMR CRC, which can be defined as a “hot tumor.” Considering the four consensus molecular subtypes (CMS) [16, 20], each with distinct molecular and immune features, dMMR CRC represents roughly 80% of the CMS1 subgroup; this is characterized by hypermutated phenotype and strong immune activation. Conversely, the immunogenic scenario changes radically in the other subgroups, shifting toward a less immunogenic TME (Fig. 1). CMS2, CMS3, and CMS4 subtypes include mainly pMMR CRC. CMS2 subtype includes 35% of CRC and is characterized by chromosomal instability (CIN) and upregulation of WNT and MYC pathways: This subtype might be defined as an “immune-excluded tumor.” The same might be said for CMS3 subtype, characterized by a high rate of K-RAS mutations and usually defined as the metabolic subgroup [21]. Recent studies have shown that CMS2 and CMS3 detain a poor immunogenic TME, with low levels of TILs and APCs inside tumor core. Therefore, in these CRC subtypes, ICIs monotherapy seems to be ineffective. A work of Jason JL et al., using The Cancer Genome Atlas (TCGA) tumor samples to evaluate a T cell-inflamed gene expression signature within different tumor types, demonstrated that tumors with activation of WNT/b-catenin signaling were characterized by a non-T cell-inflamed TME [22]. In preclinical murine models, a suppressive activity on CCL4 gene transcription by WNT/β-catenin pathway, commonly upregulated in CMS2/pMMR CRC tumors, was observed, leading to low levels of CCL4 chemokine and impaired CD103^+^ dendritic cells (DCs) and CD8^+^ T cell infiltration and subsequent activation in TME [23, 24]. Somatic mutations of K-RAS or N-RAS involve about 60% of CRC, mostly pMMR tumors, leading to a constitutive activation of the mitogen-activated protein kinase (MAPK) pathway [25]. Researchers demonstrated that KRAS mutations, common in CMS3 CRC subtype, promote an immunosuppressive TME, inducing the conversion of CD4+ cells to Tregs [26, 27•] and, by upregulation of CXCL3 expression, the main ligand of CXCR2 on MDSCs surface, support their migration inside tumor core [28]. Moreover, KRAS activation reduces tumor immunogenicity, downregulating MHC-I molecules and leading to inability of CD8+ T cells to recognize tumor cells [27•]. CMS4 subgroup, also known as “mesenchymal subgroup,” includes only 6% dMMR samples and is therefore largely represented by pMMR CRC and is characterized by strong stromal activity, angiogenesis, and TGF-β pathway activation. These tumors detain an inflamed TME, enriched of immune cells with an immunosuppressive activity such as M2 macrophages, regulatory T cells (Treg), and myeloid-derived suppressor cells (MDSCs), leading to ICIs resistance. TGF-β is involved in the control of adaptive immunity leading to the expansion of Treg and inhibition of effector T cells [29, 30]. The TGF-β pathway suppresses antigen presentation of DCs by inhibiting IFN-γ-mediated induction of the class II transactivator (CIITA) promoter, essential for MHC-II gene expression [31]. TGF-β is mostly produced by cancer-associated fibroblasts (CAFs), a group of stromal cells involved in the production of extracellular matrix components, such as collagens and fibronectin, as well as several cytokines that regulate cancer progression. In this subgroup of pMMR CRC, the higher TGF-β levels produced by CAFs lead to the exclusion of CD4+ and CD8+ T cells from the tumor center and, therefore, to ICIs inefficacy [32]. Mariathasan et al. demonstrated that the immune-excluded phenotype might be overcome using anti-Pan-TGF-b antibody, promoting T cell priming and concentration in tumor core and restoring anti PD-L1 treatment efficacy in mouse tumor models [33•]. Response to ICIs implies not only the infiltration of immune cells with a certain phenotype inside TME but also the presentation of neoantigens on tumor cells to be recognized by T cells. TMB represents the number of somatic mutations per coding area of a tumor genome and has been proposed as a predictor of response to ICIs monotherapy [34]. In this context, pMMR CRC, with a median TMB of 4 mutations/MB, is defined as a low TMB tumor [35•], with a poor load of cancer specific antigens to be detected by cytotoxic CD8^+^ T cells, further explaining ICIs inefficacy in this CRC subgroup [18, 36].

## Chemotherapy and immune checkpoint inhibitors combination

Standard chemotherapy represents the mainstay of treatment for the majority of CRC patients, either in resected stage II or III [37], and also for stage IV where polychemotherapy with 5-fluorouracil (5FU) and irinotecan, oxaliplatin, or both [38] is widely used in combination with either anti-VEGF or anti-EGFR drugs. ICIs in unselected mCRC patients usually yield poor results [39]: Since MSI-L/MSS CRC is inherently resistant to ICIs, most MSI-H patients who exhibited primary resistance to ICIs were mainly due to MSI/MMR misdiagnoses more than any other factor [40]. In lung cancer patients, a combination of ICIs and chemotherapy has been developed, improving prognosis in patients who seemed to respond poorly to ICIs monotherapy [41, 42]. Indeed, chemotherapeutic agents seem to detain immunostimulatory properties [43], and this combination strategy is currently under investigation in other tumor types [44], such as CRC. Roselli et al. [45] reported changes of peripheral blood mononuclear cell count in patients treated with FOLFIRI + bevacizumab after treatment; CD4^+^ T cells were increased, and Treg ratios were decreased as well. In patients where Treg decrease was observed, statistically significant better OS (*p* = 0.036), PFS (*p* = 0.037), and ORR (*p* = 0.0064) were seen. Van Der Kraak [46] reported that human CRC HT-116 cell lines, treated with 5FU, had a statistically significant increase in PD-L1 levels after treatment. Oxaliplatin can induce tumor cell death through a mechanism called immunogenic cell death (ICD) [47]. Therefore, its potential role as immunomodulatory agent has been widely investigated in CRC. Tesniere et al. [48] reported that oxaliplatin in CRC murine models lead to the exposure of calreticulin on membrane surface and release of high-mobility group box 1 protein (HMGB1), able to bind TLR4 of DCs and activate them [49]. Moreover, calreticulin, in addition to inhibiting angiogenesis, seems to increases TILs in TME, enhancing ICIs activity [50]. Song et al. [51] investigated the efficacy of oxaliplatin and anti-PD-L1 agent in a murine CRC model, and they showed that when oxaliplatin was added to anti-PD-L1 drug, a statistically significant reduction in tumor growth was observed compared to oxaliplatin alone. Both studies used CT26 cell line, a renowned MSS, K-RAS G12D mutated cell line [52].

Wang et al. [53] compared different sensitivity to ICIs-oxaliplatin combination in different cell lines: CT26, sensitive to ICIs, vs MC38, resistant to ICIs. This study demonstrated that MC38 cells also “regained” their sensitivity to ICIs treatment after oxaliplatin exposure. Golchin et al. [54••] also reported that oxaliplatin in combination with anti-PD-L1 was associated with improved survival and tumor control compared with oxaliplatin or anti-PD-L1 alone in CT26 tumor-bearing mice. There are several ongoing/completed trials that aim to assess the impact of chemotherapy as an immune-sensitizing agent in CRC (Table 1). The phase II POCHI trial (NCT04262687) will assess the impact of chemotherapy and ICIs in MSS CRC patients, stratified by immune cells infiltrate. Patients will receive XELOX + bevacizumab + pembrolizumab combination. Immunoscore and TuLIS score will also be performed at baseline as to evaluate their role as predictors of response to ICIs in MSS CRC patients. Finally, another phase I study (NCT03626922) will assess the impact of combination of pembrolizumab + oxaliplatin + pemetrexed in patients with MSS CRC. The study is currently ongoing.

**Table 1:** Ongoing trials of combination therapy strategies with immune checkpoint inhibitors

Identifier/reference	Study title	Study design	Population	Status	Trial description	Primary endpoints
ICIs and chemotherapy						
NCT04262687	POCHI	Phase II	First-line MSS/P-MMR CRC	Soon-to-start	XELOX + bevacizumab + pembrolizumab, single arm. All patients will be prospectively assessed by immunoscore/TuLIP score	OS
NCT03626922	-	Phase IB	First-line MSS/P-MMR CRC	Recruiting	Pemetrexed + oxaliplatin + pembrolizumab, single arm	ORR
ICIs and radiotherapy						
NCT02921256	-	Random Phase II	LARC untreated	Enrollment suspended	Randomised 3-arm treatment: standard treatment with FOLFOX-based CRT vs FOLFOX-based CRT + pembrolizumab vs FOLFOX-based CRT + veliparib + pembrolizumab	ORR
NCT04109755	PEMREC	Phase II	LARC untreated	Recruiting	Short course radiotherapy followed by pembrolizumab monotherapy	TRG grade
NCT02948348	-	Phase IB/II	LARC untreated	Recruiting	Standard CRT (with capecitabine + RT) followed by sequential nivolumab therapy	Safety/ORR
NCT04124601	CHINOREC	Phase II	LARC untreated	Recruiting	Standard CRT (with capecitabine + RT) followed by sequential nivolumab + ipilimumab therapy	Safety
NCT03921684	-	Phase II	LARC untreated	Recruiting	Standard CRT (with capecitabine + RT) followed by FOLFOX + nivolumab combination therapy	pCR rate
NCT04017455	TARZAN	Phase II	LARC untreated	Recruiting	Short course radiotherapy followed by atezolizumab + bevacizumab combination	ORR
NCT03127007	R-IMMUNE	Phase IB/II	LARC untreated	Recruiting	Standard CRT (with 5FU + RT) with concomitant atezolizumab	Safety/ORR
NCT03299660	-	Phase II	LARC untreated	Recruiting	Standard CRT (with capecitabine + RT) followed by avelumab monotherapy for 4 cycles	pCR rate
NCT03854799	AVANA	Phase II	LARC untreated	Recruiting	Standard CRT (with capecitabine + RT) with concomitant avelumab	pCR rate
ICIs and anti-angiogenic drugs						
NCT02291289	MODUL	Phase II	First-line mCRC	Active, not recruiting	Parallel cohorts each one investingating a specific biomarker-driven maintenance therapy after standard first-line. One arm investigated Atezolizumab + 5FU + bevacizumab in pMMRCRC	OS,ORR, safety
NCT02873195	BACCI	Random Phase II	First-line mCRC	Active, not recruiting	Capecitabine + bevacizumab + placebo vs capecitabine + placebo + atezolizumab	PFS (reached)
NCT04072198	NIVACOR	Phase II	First-line mCRC, RAS/BRAF mutated	Recruiting	FOLFOXIRI + bevacizumab + nivolumab, single arm	ORR
NCT03721653	AtezoTRIBE	Phase II	First-line mCRC	Active, not recruiting	FOLFOXIRI + bevacizumab + atezolizumab vs FOLFOXIRI + bevacizumab, 1:2 randomised	PFS
ICIs and anti-EGFR drugs						
NCT02713373	-	Phase I/II	Preterated EGFR naive or EGFR rechallenge candidated unresectable or mCRC	Active, not recruiting	Cetuximab + pembrolizumab, single arm	Safety/PFS/ORR
NCT03174405	AVETUX	Phase II	First-line RAS/BRAF wt mCRC	Active, not recruiting	FOLFOX + avelumab + cetuximab, single arm	PFS
NCT03608046	AVETUXIRI	Phase II	RAS wt mCRC, non-progressive disease following anti-EGFR treatment	Recruiting	Avelumab + cetuximab + irinotecan, single arm	ORR
NCT04561336	CAVE colon	Phase II	Pretreated RAS wt mCRC	Active, not recruiting	Avelumab + cetuximab, single arm	OS
NCT03442569	LCCC1632	Phase II	Pretreated RAS/RAF wt MSS mCRC	Active, not recruiting	Ipilimumab + nivolumab + panitumumab, single arm, after an initial safety lead-in cohort to ensure the three drug combination is well-tolerated	ORR
ICIS and MAPK inhibitor drugs						
NCT04185883	CodeBreak 101	Phase IB	KRAS G12C mutated advanced solid tumours	Recruiting	Sotorasib + anti PD1/MEK or other anti cancer therapies, non randomised multi arm, sequential assignement	Safety
NCT04613596	Kristal 7	Phase II	PD-L1 known advanced NSCLC	Recruiting	Adagrasib + pembrolizumab, single arm, sequential assignement	Clinical activity/ORR
NCT04044430	-	Phase I/II	Pretreated BRAF V600E mutated MSS mCRC	Recruiting	Encorafenib + binimetinib + nivolumab, single arm	ORR
NCT03668431	-	Phase II	BRAF V600E mutated mCRC	Recruiting	Dabrafenib+ trametinib + spartalizumab, single arm	ORR/safety
NCT03711058	-	Phase II	Relapsed/refractory solid tumors with expansions in MSS Colorectal Cancer	Recruiting	Copanlisib + nivolumab, single-arm non-randomised, sequential assignement	MTD/ORR
ICIS and MKI drugs						
NCT03475953	REGOMUNE	Phase I/II	Advanced or metastatic solid tumors	Recruiting	Regorafenib+ avelumab, ten cohorts considdering different solid tumors once the recommanded phase II dose (RP2D) has been determined in a 3 + 3 classical design dose escalation study	Safety/clinical activity
NCT03406871	REGONIVO	Phase I/II	Advanced or metastatic solid tumors	Active, not recruiting	Regorafenib + nivolumab, single-arm dose excalation cohort. Following dose expansion cohort considering only gastric cancer, CRC and hepatocarcinoma	RD/MTD
NCT03797326	LEAP-005	Phase II	Pretreated solid tumors	Recruiting	Pembrolizumab + lenvatinib, 2 arms multi-cohort, parallel assignement	Safety/ORR
NCT02713529	-	Phase IB/II	Pancreatic cancer, CRC, NSCLC	Active, not recruiting	AMG 820 + pembrolizumab, single arm	Safety/ORR

*ICIs* immune checkpoint inhibitors, *MSS* microsatellite stable, *P-MMR* proficient mismatch repair, *mCRC* metastatic colorectal cancer, *XELOX* capecitabine + oxaliplatin, *OS* overall survival, *PFS* progression-free survival, *ORR* objective response rate, *FOLFOX* fluorouracil + oxaliplatin, *LARC* locally advanced rectal cancer, *CRT* chemoradiotherapy, *TRG* tumour regression grade, *RT* radiotherapy, *pCR* pathological complete response, *FOLFOXIRI* fluorouracil + oxaliplatin + irinotecan, *EGFR* epidermal growth factor receptor, *MAPK* mitogen-activated protein kinase, *PD1* programmed cell death protein 1, *PD-L1* programmed death-ligand 1, *NSCLC* non-small cell lung cancer, *MTD* maximum tolerated dose, *MKI* multi-target kinase inhibitors, *RD* rational dose

## Radiotherapy and immune checkpoint inhibitors combination

Radiotherapy is rarely used in mCRC, mainly as palliative treatment of bone metastases. On the other hand, chemoradiation (CRT) is standard of treatment of locally advanced rectal cancer, either in neoadjuvant or adjuvant setting. A few papers have suggested that rectal cancer CRT might determine an increase in PD-L1 expression, thus potentially improving ICIs efficacy in this disease. Hecht et al. [55] compared PD-L1 expression between 103 pre-CRT biopsies and 159 post-CRT surgical specimens. Authors confirmed increased PD-L1 expression after CRT, particularly in the cancer invasive front (from 2.1 to 9.3%, *p* < 0.001). Low PD-L1 expression and low immune cells expression were associated with worse OS. Chen et al. [56] came to similar results: In 112 matched pre- and post-CRT locally advanced rectal cancer (LARC) biopsies, PD-L1 expression levels and tumor-infiltrating CD8+ T cells were increased after CRT. Pre- and post-CRT levels of both PD-L1 and CD8+ T-cells were associated with improved survival and reduced relapse risk. On the other hand, Shao et al. [57] reported that in patients treated with CRT, high PD-L1 expression was associated with statistically significant worse OS and higher local relapse rate, suggesting that these patients would require additional anti-PD-L1 therapy. Saigusa et al. [58] also reported that in 90 LARC patients treated with CRT, higher levels of PD-L1 levels were associated with higher risk of locoregional relapse (*p* = 0.0051). Higher levels of PD-L1 expression were associated with lower CD8+ lymphocytes. All these papers seem to suggest that CRT in LARC might cause an increase in expression of PD-L1 and also TILs in TME. On the other hand, the prognostic role of higher PD-L1 expression, particularly after CRT, seems to be less defined. As for clinical trials that will assess the real impact of radiotherapy and ICIs, a series of ongoing trials are actually been conducted (Table 1).

## Anti-VEGF and immune checkpoint inhibitors combination

There are a few published papers showing close relationship between VEGF (vascular endothelial growth factor)-driven angiogenesis and immune TME, suggesting the use of anti-VEGF treatment in combination with ICIs to overcome resistance in pMMR CRCs. Preclinical studies demonstrated that VEGF-driven angiogenesis leads to the expansion of suppressive immune cells including Tregs and MDSCs [59, 60] and increases tumor-associated macrophages’ (TAMs) infiltrates in tumor sites [61]. On the other hand, VEGF exerts its immunosuppressive effect also by the inhibition of the progenitor cells differentiation to CD4+ and CD8+ lymphocytes [62] with T cells decreased proliferation and reduced cytotoxic effects. In addition to that, VEGF has been shown to increase T cell exhaustion by increasing PD-1, CTLA-4, TIM3, and LAG3 expression on T cells. Interestingly, VEGF immunosuppressive effects have been shown to be reversible with the use of anti-VEGF drugs, providing a strong rationale for the combination of angiogenesis inhibitors and ICIs. Bevacizumab, an anti-VEGF-A antibody commonly used for mCRC treatment in combination with chemotherapy, has been evaluated in combination with ICIs in CRC in different clinical trials with conflicting results. In the phase II BACCI trial, the addition of atezolizumab to a combination of capecitabine and bevacizumab was assessed in a cohort of refractory metastatic CRC patients, providing a modest improvement in PFS (4.4 vs 3.3 months, HR = 0.72; *p* = 0.051) [63]. Another study, the MODUL trial, evaluated the addition of atezolizumab to maintenance therapy with 5FU and bevacizumab after and induction with FOLFOX and bevacizumab in patients with pMMR CRC [64] with no evidence of benefit in terms of PFS or OS. The combination of ICIs with bevacizumab and chemotherapy in first-line setting was assessed in a phase Ib trial where atezolizumab was associated with FOLFOX and bevacizumab regimen [65]. The study showed no unexpected safety signals and encouraging results in term of activity with a median PFS of 14.1 months (95% CI 8.7–17.1) and a median duration of response of 11.4 months (95% CI 7.6–15.9). The authors also demonstrated that CD8+ T-cells and PD-L1 expression were increased in tumors following administration of FOLFOX, atezolizumab, and bevacizumab. Moreover, patients with elevations in tumor-infiltrating CD8+ T cells consistent with increased expression of cytotoxic T cell signatures and PD-L1 showed sustained responses or prolonged disease control, confirming that the hypothesis that this combination could promote immune-related antitumoral activity. The use of an intensified chemotherapy regimen, such as FOLFOXIRI, in combination with bevacizumab and ICIs, is currently under evaluation in two clinical trials (ATEZOTRIBE and NIVACOR). The rationale behind this strategy relies in the observation that intensification of the chemotherapy plus bevacizumab can boost the release of novel neoantigens and infiltration of CD8+ T cells, increasing the likelihood of response to immunotherapy. Preliminary results of phase II NIVACOR trial have been recently presented [66]. The study includes patients with RAS or BRAF mutated, regardless of microsatellite status, mCRC patients treated with FOLFOXIRI and bevacizumab in association with nivolumab for eight cycles followed by maintenance treatment with bevacizumab and nivolumab. The combination was generally well tolerated with an acceptable toxicity profile and no unexpected findings. The ATEZOTRIBE is a randomized phase II trial comparing FOLFOXIRI/bevacizumab with FOLFOXIRI/bevacizumab plus atezolizumab in first-line treatment of mCRC (NCT03721653). As in the NIVACOR trial, after the induction phase, patients are treated with maintenance treatment with 5-FU and bevacizumab or the same combination in association with atezolizumab. Primary endpoint of the study is PFS (Table 1).

## Anti-EGFR drugs and immune checkpoint inhibitors combination

Cetuximab and panitumumab, monoclonal antibodies targeting epidermal growth factor receptor (EGFR), are currently used for the systemic treatment of mCRC in combination with standard chemotherapy in RAS and BRAF wild-type (wt) mCRC [67, 68]. During the last years, preclinical works have shown that immunoglobulin (Ig) G1 monoclonal antibodies (mAbs) detain a high capability for stimulating antibody-dependent cell-mediated cytotoxicity (ADCC) [69, 70•]. ADCC is an immune mechanism involving the killing of antibody-coated target cells expressing tumor-derived antigens on their surface by effector cells, usually natural killer (NK) cells [71]. Cetuximab, binding to EGFR on cancer cells and to the CD16 receptor on NK and DCs, has been shown to induce ADCC and, therefore, the secretion of pro-inflammatory cytokines (i.e., IFN-γ, TNFα) and the priming of cytotoxic T cells in the TME, stimulating immunity against tumor [72–74]. However, this initial immune stimulation is followed by the induction of immuno-suppressive mechanisms such as recruitment of Treg and MDSCs on TME and increased expression of PD-1, PD-L1, and CTLA-4 on tumor and immune cells. The synergism between anti-EGFR and ICIs might be expected on the basis of a two-step process where anti-EGFR drugs contribute to immune cell recruitment in TME and ICIs allow reactivation of immune cells that are already present; this might lead to forefront combinations in order to overcome ICIs resistance in pMMR CRC—RAS wt tumors [75]. An ongoing phase I/II trial (NCT02713373) is evaluating cetuximab and pembrolizumab combination in RAS wt mCRC, after at least one prior treatment line. Remarkably, 6 out of 9 patients achieved stable disease (SD) lasting ≥ 16 weeks, with no dose limiting toxicities (DLTs) observed [76]. The AVATUX study (NCT03174405), a phase II trial, investigated the combination of avelumab, chemotherapy (mFOLFOX6 regimen) and cetuximab in first-line RAS and BRAF wt mCRC. Preliminary results on 43 patients showed encouraging data [77]. Specifically, ORR was 79.5%, including 6 CR and 25 partial responses (PR). Moreover, 5 SD were noted; thus, DCR was 92.3%.

Phase II trial (NCT03442569) combining nivolumab and ipilimumab with panitumumab in RAS and BRAF wt pretreated MSS mCRC met its primary endpoint with a 12-week response rate of 35% [78]. In addition to these data, two clinical trials are ongoing: the AVETUXIRI trial (NCT03608046), which is investigating the efficacy of avelumab combined with cetuximab and irinotecan in patients with refractory mCRC, and the CAVE colon (2017-004392-32), a phase II study designed to evaluate the efficacy of avelumab and cetuximab in pretreated RAS WT mCRC patients (Table 1).

## MAPK pathway inhibitors and immune checkpoint inhibitors combination

MAP kinase pathway is crucial in tumor initiation and progression, and K-RAS and BRAF mutations are detected in around 30–50% and 5–10 % of CRC, respectively [79, 80]. Recent studies demonstrated in mCRC patients that molecular alterations of MAPK pathway have also immunosuppressive properties. KRAS mutations in cancer cells detain autocrine actions and impact on TME components, promoting an immunosuppressive stroma through the induction of cytokines and growth factors’ secretion [27•, 81•]. Moreover, KRAS mutations lead to upregulation of granulocyte-macrophage colony-stimulating factor (GM-CSF) in TME of CRC, enhancing the infiltration of MDSCs [82] and causing an evasion of antitumor immunity. MAPK pathway is involved in the regulation of HLA I expression, and its inhibition leads to the upregulation of HLA I molecules on cancer cell surface, facilizing recognition by CD8^+^ T cell [83–85]. Moreover, its activation on cancer cells reduces TILs, leading to tumor cell immune evasion [85, 86]. Before the discovery of K-RAS G12C isoform inhibitors AMG510 (sotorasib) and MRTX849 (adagrasib), K-RAS mutations had been considered undruggable [79]. A recent preclinical study showed the immunomodulatory properties of KRAS G12C inhibitors: After treatment with AMG510, an increased infiltration of T cells, primarily CD8+ T cells, into KRAS G12C mutated tumors in rats was observed [87••].

Phase 1/1b AMG510 CodeBreak 100 [88••] and MRTX849 Kristal-1 [89] ongoing trials have shown promising results, respectively, providing over 50 % and 90% DCR in heavily pretreated patients with KRAS G12C mutated solid tumors. Therefore, sotorasib is being studied in combination with MEK inhibitors or anti-PD-1 agents in NSCLC and CRC in CodeBreak101 [90], while Kristal-7 will provide data about adagrasib and pembrolizumab doublet in NSCLC. Even though K-RAS G12C variant is present in only 10% of all K-RAS mutations in CRC patients [79], the results of these trials might be crucial in a clinical setting where other treatment options are rather limited. Focusing on RAF/MEK axis, the combination of encorafenib + binimetinib + nivolumab and dabrafenib + trametinib + spartalizumab, an anti-PD1 drug, in patients affected by BRAF-V600E mutated pMMR CRC is being evaluated in phase I/II NCT04044430 and phase II NCT03668431 trials respectively. Preliminary data have reported 33% ORR of both combinations. Moreover, phase I/II NCT03711058 is assessing PIK3CA inhibition with copanlisib combined with nivolumab in relapsed/refractory solid tumors, including MSS CRC. Preliminary results of IMblaze370 [91], a phase III randomized trial comparing atezolizumab plus cobimetinib, a MEK1/2 inhibitor vs atezolizumab monotherapy vs regorafenib in third-line setting in pMMR CRC, are less promising. Atezolizumab + cobimetinib regimen had 8.87 months mPFS vs 7.10 months for atezolizumab alone vs 8.51 months for regorafenib. Moreover, the combined treatment arm with atezolizumab + cobimetinib was associated with a significantly higher (64%) grade 3–4 adverse events’ rate compared with the other study arms (Table 1).

## Multi-target kinase inhibitors and immune checkpoint inhibitors combination

TAMs are involved in cancer progression inducing angiogenesis through VEGF secretion, immune evasion through immunosuppressive cytokines release (IL-10, TGFβ), and upregulating immune checkpoint expression such as PD-L1 on tumor cells [92–95]. Regorafenib is a multi-target kinase inhibitor (MKI) that not only inhibits VEGF and its receptor VEGFR but also other pro-angiogenetic molecules, highly expressed by TAMs, such as EGF homology domain 2 (TIE2), blocking their recruitment into the TME [96]. Therefore, combinations of ICIs and regorafenib or lenvatinib, another MKI, are currently under investigation. Regorafenib plus nivolumab combination [97] showed 40% ORR in the phase Ib REGONIVO study in both gastric and colorectal cancer cohorts. Thirty-six percent of ORR was observed in the CRC cohort alone with 7.9 mPFS in pMMR tumors. Phase II REGOMUNE trial [98] with a regorafenib and avelumab combination reported 53.3% SD rate; however, no objective responses were seen. Although median PFS and median OS were quite modest (3.6 and 10.8 months respectively), pathological review of tumor samples showed that a significant increase in CD8^+^ T cells infiltrates in 60% of cases and this change was associated with improved outcome, suggesting a synergism in shaping the TME of this drug combination. Conversely, higher baseline TAMs infiltration was significantly associated with shorter PFS. Combination of ICIs and lenvatinib (NCT03797326) is under investigation in a phase II study in patients with previously treated advanced solid tumors, including pMMR CRC. This study was based also on the results of the LEMON trial [99], a phase II study that showed 70% DCR rate of lenvatinib monotherapy in patients with mCRC refractory to standard chemotherapy. Another phase Ib/II trial (NCT02713529) will assess the combination of pembrolizumab with AMG 820, an antibody targeting colony-stimulating factor 1 receptor (CSF-1R) that regulates TAMs recruitment and survival; preliminary data of this study have reported only 4.9% ORR in pMMR CRC patients’ cohort (Table 1).

## Conclusions

After the discovery of the effectiveness of ICIs in several tumor types such as melanoma, lung cancer, and kidney cancer, researchers have investigated the activity of these drugs also other solid tumors. In gastrointestinal malignancies, most studies have been met with disappointment, with the notable exception of patients with MSI-H-dMMR subset. As it stands, approximately 95% of mCRC patients, represented by those with pMMR mCRC tumors, remain outside from this promising therapeutic opportunity. On the second thought, lack of activity of ICIs monotherapy in majority of mCRC should not be surprising, based on the wide genetic and epigenetic heterogeneity associated with different types of CRC. Starting from CMS classification, as previously discussed, each CMS detains its own molecular identity from which derives the immunogenicity of the tumor itself. As it can be seen, most promising ways to introduce ICIs in clinical setting for majority of patients with mCRC are those where these drugs will be combined with other treatment options. Most trials have focused on biological rationale of new drug combinations that might turn what is usually considered from an immunogenic point of view as “cold” tumor into a “hot” tumor.

What seems to be lacking in all these trials is a prospectively defined translational research assessment (with a few notable exceptions): It is eagerly awaited that based on the innovative mechanism of action of ICIs compared with standard chemotherapy, previous “errors” that were committed during the development of anti-VEGF and anti-EGFR-based therapy might be overcome by taking into account pre-specified molecular stratification of these patients.
